# Public sector low threshold office-based buprenorphine treatment: outcomes at year 7

**DOI:** 10.1186/s13722-017-0072-2

**Published:** 2017-02-28

**Authors:** Elenore Patterson Bhatraju, Ellie Grossman, Babak Tofighi, Jennifer McNeely, Danae DiRocco, Mara Flannery, Ann Garment, Keith Goldfeld, Marc N. Gourevitch, Joshua D. Lee

**Affiliations:** 1 Department of Population Health, NYU School of Medicine, 227 East 30th St, New York, NY 10016 USA; 2 Department of Medicine, Division of General Internal Medicine and Clinical Innovation, NYU School of Medicine, New York, NY USA; 3 Department of Population Health, NYU School of Medicine, 227 East 30th St #712, New York, NY 10016 USA

**Keywords:** Buprenorphine, Induction, Primary care, Office-based treatment, Opioid dependence

## Abstract

**Background:**

Buprenorphine maintenance for opioid dependence remains of limited availability among underserved populations, despite increases in US opioid misuse and overdose deaths. Low threshold primary care treatment models including the use of unobserved, “home,” buprenorphine induction may simplify initiation of care and improve access. Unobserved induction and long-term treatment outcomes have not been reported recently among large, naturalistic cohorts treated in low threshold safety net primary care settings.

**Methods:**

This prospective clinical registry cohort design estimated rates of induction-related adverse events, treatment retention, and urine opioid results for opioid dependent adults offered buprenorphine maintenance in a New York City public hospital primary care office-based practice from 2006 to 2013. This clinic relied on typical ambulatory care individual provider-patient visits, prescribed unobserved induction exclusively, saw patients no more than weekly, and did not require additional psychosocial treatment. Unobserved induction consisted of an in-person screening and diagnostic visit followed by a 1-week buprenorphine written prescription, with pamphlet, and telephone support. Primary outcomes analyzed were rates of induction-related adverse events (AE), week 1 drop-out, and long-term treatment retention. Factors associated with treatment retention were examined using a Cox proportional hazard model among inductions and all patients. Secondary outcomes included overall clinic retention, buprenorphine dosages, and urine sample results.

**Results:**

Of the 485 total patients in our registry, 306 were inducted, and 179 were transfers already on buprenorphine. Post-induction (n = 306), week 1 drop-out was 17%. Rates of any induction-related AE were 12%; serious adverse events, 0%; precipitated withdrawal, 3%; prolonged withdrawal, 4%. Treatment retention was a median 38 weeks (range 0–320) for inductions, compared to 110 (0–354) weeks for transfers and 57 for the entire clinic population. Older age, later years of first clinic visit (vs. 2006–2007), and baseline heroin abstinence were associated with increased treatment retention overall.

**Conclusions:**

Unobserved “home” buprenorphine induction in a public sector primary care setting appeared a feasible and safe clinical practice. Post-induction treatment retention of a median 38 weeks was in line with previous naturalistic studies of real-world office-based opioid treatment. Low threshold treatment protocols, as compared to national guidelines, may compliment recently increased prescriber patient limits and expand access to buprenorphine among public sector opioid use disorder patients.

## Background

Buprenorphine, approved for office-based treatment of opioid use disorders (opioid dependence) by waivered prescribers, has become the cornerstone of opioid treatment in the US [1–3]. Consistent with a chronic disease model, long-term buprenorphine maintenance has been shown superior to short-term tapers or time-limited treatment windows [4, 5]. However, too few patients overall access buprenorphine, particularly in rural areas and among the Medicaid-insured and underserved [6–8]. A 2013 New York City survey of buprenorphine-waivered physicians estimated that only 10% accepted Medicaid, meaning higher prescriber patient ‘caps’ and Medicaid expansion theoretically only impact a small proportion of the total providers who serve public sector patients [9]. We surveyed N = 72 NYC public sector buprenorphine providers in 2016 regarding barriers to prescribing; time and resource constraints were among the most heavily agreed to barriers to buprenorphine practice [10]. Per multiple provider survey studies conducted since buprenorphine’s 2002 US approval, limited clinician time, office space, support staff, reimbursement, and induction logistics are barriers to prescribing [11–15].

These familiar buprenorphine practice barriers reflect ‘high threshold’ practice standards, as defined by somewhat dated but still current Substance Abuse and Mental Health Service Administration (SAMHSA) office-based buprenorphine guidelines, the Treatment Improvement Protocol (TIP) 40, Clinical Guidelines for the Use of Buprenorphine in the Treatment of Opioid Addiction [1, 2]. TIP40 remains the basis of mandatory buprenorphine-waiver training courses. These guidelines, as well as buprenorphine product labels, endorse only in-office, observed buprenorphine induction during a potentially prolonged office visit, recommend daily or frequent follow-up immediately post-induction, and heavily emphasize ancillary psychosocial counseling concurrent with pharmacotherapy medical management (Table 1) [16]. TIP40 has presumably heavily influenced payer policies, such as buprenorphine prior authorization (PA) criteria. One current New York State payer’s PA, for example, requires that a buprenorphine patient be enrolled in “substance abuse rehabilitation services,” in addition to an individual prescriber’s practice [17]. More recent consensus guidelines from the American Society of Addiction Medicine (2015) have evolved and arguably liberalized some of the original TIP40 recommendations regarding observed versus unobserved induction, the timing of immediate post-induction observation, and recommendations for additional psychosocial counseling [3]. The 2015 ASAM guidelines, however, preserve many of the relatively intensive office-based protocols requiring more space, time, and effort than may be needed for safe and effective treatment, including routine use of observed induction, reserving unobserved induction only for experienced providers or patients, and a blanket endorsement of additional psychosocial counseling, particularly for new buprenorphine patients.

**Table 1:** Buprenorphine practice guidelines and low threshold office-based protocols

Source	Induction	Follow-up	Counseling
Center for Substance Abuse Treatment, Treatment Improvement Protocol (TIP) 40, Clinical Guidelines for the Use of Buprenorphine in the Treatment of Opioid Addiction [1]	The consensus panel recommends that physicians administer initial induction doses as observed treatment (e.g., in the office); further doses may be provided via prescription thereafter. This ensures that the amount of buprenorphine located in the physician’s office is kept to a minimum. Following the initial buprenorphine dose, patients should be observed in the physician’s office for up to 2 hours…Before the initial buprenorphine induction dose…the patient should preferably be exhibiting early signs of opioid withdrawal (e.g., sweating, yawning, rhinorrhea, lacrimation). (p.52)	Induction Day 2 and Forward: Patient returns to office on buprenorphine/naloxone (Figure 4-2)…Patients who return on Day 2 experiencing withdrawal symptoms should receive an initial dose of buprenorphine/naloxone equivalent to the total amount of buprenorphine/naloxone…administered on Day 1 plus an additional 4/1 mg (maximum initial dose of 12/3 mg). If withdrawal symptoms are still present 2 hours after the dose, an additional 4/1 mg dose can be administered. (pp.54–56)	Pharmacotherapy alone is rarely sufficient treatment for drug addiction. For most patients, drug abuse counseling—individual or group—and participation in self-help programs are necessary components of comprehensive addiction care. As part of training in the treatment of opioid addiction, physicians should at a minimum obtain some knowledge about the basic principles of brief intervention in case of relapse. Physicians considering providing opioid addiction care should ensure that they are capable of providing psychosocial services, either in their own practices or through referrals to reputable behavioral health practitioners in their communities. (Executive Summary XX)
American Society of Addiction Medicine (ASAM) National Practice Guideline for the Use of Medications in the Treatment of Addiction Involving Opioid Use (2015) [3]	(3) Clinicians should observe patients in their offices during [buprenorphine] induction. Emerging research, however, suggests that many patients need ‘‘not’’ [sic] be observed and that home buprenorphine induction may be considered. Home based induction is recommended only if the patient or prescribing physician is experienced with the use of buprenorphine. This is based on the consensus opinion of the Guideline Committee. *Induction* Induction within the clinician’s office is recommended to reduce the risk of precipitated opioid withdrawal. Office-based induction is also recommended if the patient or physician is unfamiliar with buprenorphine. However, buprenorphine induction may be done by patients within their own homes.^84^ Home-based induction is recommended only if the patient or prescribing physician is experienced with the use of buprenorphine. The recommendation supporting home induction is based on the consensus opinion of the Guideline Committee. (p. 33)	(8) Patients should be seen frequently at the beginning of their treatment. Weekly visits (at least) are recommended until patients are determined to be stable. There is no recommended time limit for treatment. *Monitoring treatment* Patients should be seen frequently at the beginning of their treatment. Weekly visits (at least) are recommended until patients are determined to be stable. The stability of a patient is determined by an individual clinician based on a number of indicators which may include abstinence from illicit drugs, participation in psychosocial treatment and other recovery based activities, and good occupational and social functioning. Stable patients can be seen less frequently but should be seen at least monthly. (p. 34)	(5) Psychosocial treatment should be implemented in conjunction with the use of buprenorphine in the treatment of opioid use disorder. Psychosocial treatment and treatment with buprenorphine clinicians who are prescribing buprenorphine should consider providing or recommending office-based or community-based psychosocial treatment. There is some research evidence that the addition of psychosocial treatment improves adherence and retention in treatment with buprenorphine63,94,95; however, these findings are mixed.29,96–99 It is recommended that clinicians offer patients psychosocial treatment early in their treatment with buprenorphine. Effective therapies may include the following: (1) cognitive behavioral therapies; (2) contingency management; (3) relapse prevention; and (4) motivational interviewing. (p. 39)
Low Threshold Primary Care Office-based Buprenorphine Treatment	Unobserved induction only; no in-person or in-clinic induction. Patient handout written and text-message or phone support as needed.	Weekly to monthly or less than monthly, varies per patient. Typically, a new induction patient is seen one-week following induction, then less frequently. Refills and less than monthly follow-up are allowed for stable patients.	Generally endorsed by providers for all patients; 12-step and other counseling involvement assessed at follow-up; no requirement or mandate for any additional counseling; no additional counseling available in-clinic.

Since 2002, many buprenorphine providers in public sector, general care, HIV, and harm reduction clinical settings have customized leaner, lower threshold buprenorphine practices, which seek to provide quality care while working in resource-constrained settings [18–24]. Our own office-based primary care clinic in a New York City public hospital is characterized by the following: predominantly Medicaid or uninsured patients, universal unobserved buprenorphine induction following an initial new patient visit and a diagnosis of opioid dependence, weekly then less frequent follow-up, and a general recommendation but no requirement or mandate for additional psychosocial treatment, which has otherwise not been available in our clinic [25]. During unobserved or “home” buprenorphine induction, the patient is diagnosed with opioid dependence during their initial office visit, offered a buprenorphine prescription, provided with instructions to self-administer initial induction and maintenance doses after leaving clinic and when experiencing opioid withdrawal symptoms, and instructed to return at a later time [26]. Subsequent follow-up visits occur at our clinic weekly, then bi-weekly, then monthly or less frequently among stable patients. While we encourage all of our patients to access additional community treatment, counseling and 12-step resources, these are not mandates; psychosocial support is primarily delivered during the provider-patient medical management visit, which is structured as a standard ambulatory care follow-up visit.

We have offered this version of low threshold office-based buprenorphine treatment since 2006 in a New York City Public Hospital adult primary care clinic, but have not examined overall or long-term (12+ months) retention, or factors associated with time in treatment, including unobserved induction outcomes. Long-term retention in office-based buprenorphine treatment has not been widely studied; other sites have reported around 50–60% retention at 12-months [18, 24], and 38% at 24-months among patients initially successful in treatment [27]. This prospective cohort study analyzed a consecutive registry of buprenorphine patients treated in our practice over a 7-year period, 2006–2013, and tracked patient characteristics, induction outcomes, and treatment retention.

## Methods

The clinical site, populations, and clinic and assessment procedures for initial and follow-up visits, unobserved buprenorphine induction, and data collection have been previously described and are briefly summarized here [25].

### Site

Since August 2006, the Bellevue Hospital Adult Primary Care Center, a large urban-public hospital, has offered office-based buprenorphine treatment for opioid dependent adults. A physician team comprised of 5–6 Internal Medicine physicians, of whom 3 were certified in Addiction Medicine, co-managed a buprenorphine patient panel during two weekly half-day sessions. Physicians were generally available for off-schedule in-clinic consultations for any urgent matters during regular clinic hours and by phone at all times.

### Population

The clinic offered office-based induction and maintenance treatment to opioid dependent adults age 18 years or older. Referral sources included detoxification units, chemical dependency outpatient programs, the criminal justice system, other primary care providers, and patient word-of-mouth.

### Initial induction visit

Buprenorphine treatment was offered following a clinical diagnosis of opioid dependence per Diagnostic and Statistics Manual-IV and product labels, and a co-signed physician-patient office-based buprenorphine treatment plan. Physicians assessed medical, psychiatric, and substance use diagnoses during a 30–45-minute new patient appointment. Urine toxicology assays were mandatory. Hepatitis C and HIV screening was encouraged throughout treatment but not mandated at baseline; similarly, liver serologies were obtained when indicated and not mandated at baseline.

A bilingual pictogram-based pamphlet instructed patients step by step on unobserved induction using a “teach-back” method. Initial buprenorphine prescriptions for both induction and transfer-in patients (patients already on a stable buprenorphine dose but new to our clinic) were written for seven days, usually for fourteen 8-mg/2-mg tablets, with follow-up in one week. This induction pictogram is available as an on-line resource as part of a prior manuscript [25]. Induction and maintenance was undertaken exclusively with prescription of the combination buprenorphine-naloxone products; the exception being pregnant patients, who were prescribed buprenorphine monotherapy (Subutex).

### Follow-up visits

Standard 20-min ambulatory visits occurred every 1–4 weeks during the first few months of treatment. Patients stable on maintenance doses were seen at 4–16 week intervals thereafter. Shorter visit intervals were scheduled if illicit opioid, other drug misuse or other problematic behaviors or safety concerns persisted. A Medical Management treatment platform counseled around treatment goals including illicit opioid abstinence and improved functional status, medication side effects and dosing, encouragement of ancillary drug treatment, counseling or 12-step engagement, and addressed primary medical care and health maintenance issues. Maintenance dosing ranged from 2–32 mg/day until around 2009–2010, when New York State Medicaid dispensing limits and evolving clinical experience consolidated around a 2–24 mg/day maintenance dose range. Urine toxicology was obtained at each visit. Psychiatrically co-morbid patients were referred to on-site psychiatric services. If patients were repeatedly unable to keep scheduled appointments, unusually disruptive or threatening, highly likely to be diverting buprenorphine, or had continued uncontrolled opioid or other drug use that was deemed unsafe for continued controlled substance prescribing and office-based treatment, the buprenorphine dose would be tapered and the patient referred to a higher level of care, including a within-hospital opioid treatment program.

### Data collection

All individual patients receiving a buprenorphine prescription from August 2006 through June 31, 2013 were included in a clinical registry. An initial clinical assessment documented demographic characteristics, lifetime substance use, and addiction treatment histories. Follow-up assessments documented treatment outcomes (i.e., induction adverse events, opioid and other drug misuse by self-report and urine toxicology). This data, in addition to visit attendance and prescription records, were periodically extracted from the electronic medical record to compile a registry dataset. Retrospective chart review was conducted in cases of incomplete or missing data. The dataset was de-identified for analysis and managed using Research Electronic Data Capture (REDCap). The New York University School of Medicine Institutional Review Board approved this registry protocol.

### Outcomes and analysis

The subset of patients presenting for buprenorphine induction (versus those transferring their care, already on buprenorphine maintenance by self-report) was identified. Baseline characteristics were summarized using descriptive statistics. Primary outcomes of interest were follow-up rates at 1-week post-induction and rates of adverse events (AEs) and serious adverse events (SAEs). AEs were classified into four categories: 1) precipitated opioid withdrawal, defined as any sudden onset or worsening of opioid withdrawal symptoms following the initial dose of buprenorphine, 2) protracted opioid withdrawal, defined as opioid withdrawal symptoms that began before induction and persisted until or past day 2 of treatment, 3) SAEs, defined as death, a life-threatening or other event necessitating emergency medical treatment, hospitalization, or persistent or significant disability or incapacity, and 4) other adverse events. Bivariate logistic regression estimated associations of prior buprenorphine experience and methadone-to-buprenorphine, two baseline variables of interest, with any induction-related AE.

Treatment retention was analyzed as a continuous variable (weeks) consisting of the time between the initial and last week of the last active buprenorphine maintenance or taper prescription. Kaplan-Meyer survival curves displayed overall retention among the total clinic cohort, inductions, and transfers. Cox proportional hazard models, among the total clinic cohort and among inductions, examined associations with retention. We excluded from this survival analysis n = 8 patients with mean gaps in treatment of > 18 weeks, indicating sporadic and in effect multiple treatment episodes rather than a continuous single treatment episode. We described several outcomes across the total clinic cohort, including buprenorphine maintenance dose levels (median, range) and rates of positive urine toxicology samples, which were summarized as overall group means across time in treatment.

## Results

From August 2006 through June 2013, 485 patients were prescribed buprenorphine, 306 of these were inducted, and 179 transfers were already on buprenorphine from a previous provider (Table 2). Induction patients were primarily male, Medicaid-insured, and most (68%) had previous experience taking buprenorphine (either licit or illicit). Nearly all (92%) were primarily using and dependent on heroin (vs. prescription opioids), used intranasally, and were current tobacco smokers (82%). Compared to transfers, induction patients included a higher rate of homelessness (defined as shelter or street homeless) and were more likely African American (vs. White).

**Table 2:** Baseline patient characteristics

	All patients (N = 485) n (%)	Inductions^a^ (n = 306) n (%)	Transfers^a^ (n = 179) n (%)
Mal	402 (83)	256 (84)	146 (82)
Age, average (range)	47 (23–73)	47 (24–71)	47 (24–73)
Race and ethnicity^b^			
Black	140 (29)	107 (35)*	33 (18)
Hispanic	60 (12)	38 (12)	22 (12)
White	147 (30)	76 (25)*	71 (40)
Insurance status^c^			
Medicaid	294 (61)	193 (64)	101 (56)
Commercial	39 (8)	15 (5)*	24 (13)
Medicare	11 (2.5)	6 (2)	5 (3)
Uninsured/self pay	113 (23)	71 (22)	42 (24)
Unemployed			
Homeless (shelter or street)^d^	80 (16)	64 (21)*	16 (9)
History of Incarceration^e^	338 (70)	227 (74)*	111 (62)
Hepatitis C positive, self-report	152 (31)	95 (30)	57 (40)
HIV positive, self-report	47 (10)	32 (10)	15 (10)
Opioid use			
Heroin use, last 7 days	252 (52)	220 (72)*	32 (18)
Heroin use, lifetime	444 (92)	289 (94)*	155 (87)
Prescription opioid misuse, last 7 days	105 (22)	88 (29)*	17 (10)
Prescription opioid Misuse, lifetime	274 (56)	174 (57)	100 (56)
IV drug use, last 7 days	104 (22)	88 (29)*	16 (9)
Buprenorphine, previous illicit or licit use	368 (76)	199 (65)*	179 (100)
Methadone maintenance, previous	295 (61)	191 (62)	104 (63)
Methadone maintenance, current	26 (7)	26 (8)*	0 (0)
Other drug use			
Cocaine use, last 7 days	102 (21)	76 (25)*	26 (15)
Benzodiazepine use, last 7 days	57 (12)	36 (12)	21 (12)
Cannabis use, last 7 days	83 (17)	53 (17)	30 (18)
Heavy drinking (>5 drinks per occasion), last 12 months	125 (26)	85 (28)	40 (24)
Smoking, current	391 (81)	252 (82)	139 (78)

* Indicates baseline demographics that are statistically different (<0.05) between induction patients and patients transferring care, using Fisher’s exact test or Chi square test of independence

^a^Inductions refer to patients offered a new buprenorphine induction prescription. Transfers were existing buprenorphine patients transferring care to our practice and induced elsewhere onto buprenorphine

^b^ Missing data: all patients (n = 138); inductions (n = 85); transfers (n = 53)

^c^Missing data: all patients (n = 280), inductions (n = 19), transfers (n = 7)

^d^Missing data: all patients (n = 18), inductions (9), transfers (n = 9)

^e^Missing data: all patients (n = 85), inductions (46), transfers (n = 39)

Unobserved induction outcomes included week 1 retention of 83% (Table 3). There were a total of 38 AE reported (12% of 306 total inductions; 15% of 254 retained at week 1). Eleven (3% of 306) patients reported precipitated withdrawal; 14 (5% of 306) prolonged withdrawal. No SAEs were observed or reported. Prior buprenorphine experience and methadone-to-buprenorphine inductions were not associated with induction AE (data not shown).

**Table 3:** Unobserved induction outcomes

Induction outcome	n (%)
Unobserved induction cases	305 (100)
Lost to follow-up at week 1	52 (17)
≥1 induction-related adverse event (AE)	38 (13)
Precipitated withdrawal	10 (3)
Prolonged withdrawal	13 (4)
Serious adverse event (SAE)	0 (0)
Other induction-related AE^a^	15 (5)

^a^Other reported AEs included likely induction-related complaints not consistent with precipitated or prolonged withdrawal syndromes

**Fig. 1:**
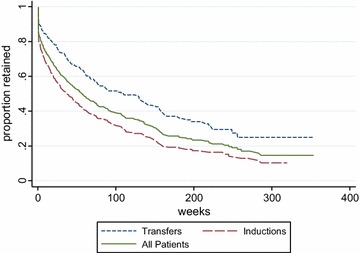
Kaplan Meyer survival curves: retention in treatment: all patients (N = 477), inductions (n = 302), and transfers (n = 175). Excludes n = 8 participants with >18 week gaps between visits: 4 inductions, 4 transfers

Treatment retention was a median of 38 weeks (range 0–320 weeks) among induction patients (n = 302); 110 weeks (0–353 weeks) among transfers (n = 175), and 57 weeks for all patients (n = 477) (Fig. 1). Among inductions, 25% dropped out by week 5, 50% by week 38, and 75% by week 144, or around 3 years following an initial visit. Factors associated with shorter time to drop-out in adjusted Cox proportional hazard models were, among the entire clinic population: age, some later years of first clinic visit versus a combined 2006–2007 ‘reference year’, induction (vs. transfer), and active heroin use (Table 4). Among inductions only, no factor was significantly associated with time to drop-out in an adjusted model, including prior buprenorphine experience, or induction-related adverse events. Reporting other outpatient counseling of 12-step involvement at baseline was not associated with time to drop-out in all patients or inductions.

**Table 4:** Factors associated with drop-out (fewer weeks in treatment), Cox proportional hazard models

Baseline or induction-related characteristic	All patients, n = 477				Inductions, n = 302			
	Hazard ratio	95% CI	Adjusted^a^ HR	95% CI	Hazard ratio	95% CI	Adjusted^b^ HR	95% CI
Age (increasing by year)	0.99	0.97–1.00	0.98	0.97–1.00	0.99	0.97–1.00	0.99	0.97–1.00
Unemployed	0.87	0.68–1.10	–	–	0.72	0.54–0.95	0.74	0.54–1.01
Year of First Visit (ref. 2007)								
2008	0.66	0.45–0.96	0.81	0.54–1.20	1.02	0.63–1.66	1.10	0.62–1.96
2009	0.65	0.48–0.88	0.67	0.49–0.91	0.78	0.55–1.11	0.94	0.64–1.38
2010	0.75	0.55–1.04	0.75	0.54–1.04	0.72	0.48–1.07	0.88	0.57–1.35
2011	0.67	0.47–0.97	0.70	0.49–1.01	0.83	0.54–1.28	1.00	0.63–1.58
2012	0.62	0.36–1.06	0.61	0.35–1.05	0.61	0.31–1.22	0.67	0.33–1.36
2013	0.64	0.29–1.38	0.62	0.29–1.34	0.60	0.24–1.50	0.70	0.28–1.78
Inducted	1.71	1.36–2.16	1.46	1.10–1.93	–	–	–	–
Prior buprenorphine	1.29	0.49–0.82	–	–	0.79	0.60–1.04	0.79	0.58–1.06
Heroin use, active	1.59	1.27–1.99	1.25	0.96–1.64	1.20	0.89–1.61	1.27	0.93–1.75
Cocaine use, active	1.32	1.03–1.71	1.18	0.90–1.54	1.22	0.91–1.63	1.15	0.83–1.59
Outpatient counseling, active	0.97	0.72–1.31	–	–	0.81	0.54–1.21	–	–
12-step attendance, active	1.01	0.79–1.29	–	–	1.02	0.77–1.37	–	–
Any induction-related AE	–	–	–	–	1.24	0.84–1.81	–	–
Methadone-to-buprenorphine induction	–	–	–	–	1.02	0.64–1.64	–	–

Factors not shown and not significantly associated with retention among all patients or inductions: gender, ethnicity, homelessness, uninsured, active benzodiazepine or cannabis use

^a^Hazard model adjusted for age, year of first visit, inducted, active baseline heroin and cocaine use. All transfer patients had prior buprenorphine experience; adding this term to the model reduces the significance of induction and vice versa

^b^Hazard model adjusted for age, unemployment, year of first visit, prior buprenorphine experience, active baseline heroin and cocaine use

The median dose of buprenorphine, calculated from the last prescribed maintenance dose for each patient, was 16 mg (range 1–32 mg; mean, 18 mg). Overall, there was a mean opiate positive urine toxicology rate of 40% across all treatment visits. Rates were 24% for cocaine and 17% for benzodiazepines. Opiate positive urine rates declined with longer treatment retention: among patients who were in treatment <12 weeks, the rate of opiate positive urines was 60%; >24 weeks, 30%; and >52 weeks, 27% (*p* < 0.001).

## Discussion

This naturalistic registry study examined up to 7 years of treatment retention among underserved opioid dependent adults receiving buprenorphine maintenance in a low threshold, public hospital, office-based primary care setting. This included 306 new patients exclusively initiated onto buprenorphine by unobserved induction. A typical new induction patient during 2006–2013 was a Medicaid-insured, heroin-dependent adult male who uneventfully began buprenorphine treatment, remained in maintenance for about 9 months, and saw diminishing rates of opioid misuse as time in treatment lengthened. Long-term retention among new induction patients was comparable to previous studies from other centers, with over half retained in treatment at 24 weeks and 20% retained for greater than 3 years [18, 27]. Including transfers already established on buprenorphine at treatment episode entry, the median time in treatment for any new patient was 110 weeks, or over 2 years of follow-up among half of our patients.

These results compare favorably to usual retention and adherence rates for patients with other chronic conditions, such as hypertension, diabetes, or HIV; prevalent conditions which are in fact quite difficult to adequately control and regularly monitor in a majority of patients within a typical public sector primary care practice across extended periods of time [28–30]. A small number of our transfer patients were in early buprenorphine clinical trials, and have maintained on buprenorphine since before FDA approval in 2002. Long-term, presumably indefinite, office-based buprenorphine maintenance appears an important and sustainable treatment outcome for a substantial proportion of patients. In a recent long-term follow-up survey of former participants in a prescription opioid addiction buprenorphine treatment trial, 37% of available participants surveyed at Month 42 post-randomization reported continued opioid agonist maintenance [31]. In our sample as in others, longer time in treatment corresponded to higher rates of heroin and other opioid abstinence.

Unobserved induction outcomes reported here add to a now sizable body of literature demonstrating overall feasibility/acceptability and low rates of complications associated with unobserved buprenorphine induction [21, 26, 32, 33]. There were no SAEs reported from the patients for whom we had post-induction follow up and low rates of precipitated withdrawal. Precipitated withdrawal appears to occur in about 5–10% of inductions, regardless of unobserved or observed induction approaches [34–36]. Among week 1 dropouts, there were likely additional cases of precipitated withdrawal or SAEs. Previously, however, we have shown that buprenorphine patients dropping out at week 1 but providing follow-up by phone were very likely to have had insurance or self-pay issues which prevented filling the initial buprenorphine-naloxone prescription and any induction [25]. Rates of week 1 drop-out range from 6 to 28% in the literature, and appear to occur regardless of observed versus unobserved approaches [37–39].

Among inductions, no single baseline factor predicted longer retention (time-in-treatment). Analyzing the entire cohort of induction and transfer patients, young, active heroin users had the shortest retention. Several factors did not associate with differential retention as expected, including previous buprenorphine experience and induction-related adverse events [40]. Patient self-report of previous experience with buprenorphine became increasingly common: 84% from 2008 to 2013 versus 37% during 2006–2007. As prior buprenorphine experience among new patients grew, initial associations with retention likely diminished. This same trend towards widespread experience with buprenorphine among out-of-treatment opioid dependent individuals also likely explained mush about the relative ease and safety of unobserved induction over time. Patients increasingly had already taken, used, and experienced buprenorphine safely and effectively on their own. Active heroin use, versus non-heroin opioid use or transferring patients already relatively stable on buprenorphine, and younger age, are both familiar risk factors for worse opioid treatment outcomes, including treatment drop-out [41–43]. Uninsured versus active health insurance did not affect retention, likely due to the New York City Health + Hospital Corporation’s outpatient pharmacy policies, which supported buprenorphine maintenance for all patients regardless of their ability to pay. Pharmacy benefits and health insurance coverage are crucial components of adequate chronic disease management.

Higher threshold, guideline-based new buprenorphine patient protocols, consisting of observed induction, as much as daily initial in-clinic follow-up post-induction, and requirements or strong recommendations for additional psychosocial counseling, are arguably idealized versions of traditional opioid treatment programs or intensive outpatient models of care, and may limit overall prescribing. Further, these guideline-based recommendations are not clearly evidence-based, as with unobserved versus observed induction. While the field lacks a large, definitive randomized trial of observed versus unobserved induction, there is to date no evidence from any clinical trials or observational study supporting any particular buprenorphine induction approach as superior or inferior in terms of safety, retention, or opioid abstinence [26, 32, 33]. The weight of the evidence from observational, non-randomized trials points to the two induction approaches as being essentially equivalent and the outcomes the same regardless of patient characteristics or provider experience. A recent large emergency department buprenorphine initiation randomized trial, for instance, adopted unobserved induction in a majority (57%, 65 of 114) of participants randomized to buprenorphine safely and by necessity; it was not practical to hold and observe many ED patients newly diagnosed with an opioid disorder and appropriate for buprenorphine induction long enough to complete an observed induction in the ED [44]. Regarding additional psychosocial counseling for new or established buprenorphine patients, which is not available in a typical primary care practice, the evidence to date is explicitly clear that additional counseling plus office-based buprenorphine is ineffective at improving retention, taper success/failure, or rates of illicit opioid use compared to office-based medical management alone [37, 45–48]. Baseline involvement in other outpatient behavioral counseling or 12-step did not correlate with longer retention in this study. A recent related analysis of pre-treatment 12-step involvement and in-treatment 12-step mandates for buprenorphine patients showed some associations with longer retention and pre-treatment involvement, but no association with retention and in-treatment 12-step mandates [49].

Lower versus higher threshold office-based practice, or unobserved versus observed induction, essentially appear to be a matter of clinician and patient preferences and available clinical resources. Either induction approach seems to allow for new patients to induct onto buprenorphine safely and effectively. In-office observed induction is by definition more logistically, time- and resource-intensive, and is repeatedly cited as a barrier to buprenorphine prescribing. While the most recent ASAM guidelines endorse unobserved induction among experienced providers and patients, unobserved induction should arguably be the default induction protocol for most patients and providers, if the overall goal is to rapidly and substantially increase the number of active office-based providers and patients. This has been the case in France, which implemented general practitioner-prescribed buprenorphine maintenance as a public health approach to heroin addiction and overdose deaths, without guidelines requiring in-office observed induction or mandating psychosocial counseling involvement [50]. The US buprenorphine X-waiver training and registration requirements, increasing but still limited patient caps, and these historically conservative treatment guidelines favoring higher-threshold models of care; all of these arguably limit access to buprenorphine. US buprenorphine restrictions remain in stark contrast to the routine use of any other schedule II or III controlled substance, including opioid analgesics and benzodiazepines. Reducing barriers to buprenorphine access is now a national public health priority. Low threshold practice models have an obvious and important role to play in a successful expansion.

### Limitations

This study has important limitations. The clinical protocols described were not contrasted with comparison conditions or interventions. Our clinic only offered unobserved inductions, weekly or less frequent follow-up with physician prescribers, and mandated or offered no in-clinic ancillary counseling. The registry cohort design lacked any observed induction or other more intensive control condition. Results do not demonstrate that unobserved induction is equally safe, effective, equivalent, or non-inferior versus unobserved induction. Outcomes were derived from clinical assessments and the electronic medical record, including patient-reported induction adverse events and retention (time to drop-out). Retention estimates included periods of missed visits and delayed prescription refills, which are common in many patients. While we restricted the survival analysis to patients with 18 weeks or less between any two visits, we did not otherwise control for shorter gaps in care. This likely underestimated the extent to which adherence to daily buprenorphine maintenance can be intermittent and episodic in many patients, as possibly evidenced by the 27% positive opioid urine rate among all patients retained in treatment for one year or longer.

## Conclusion

This longitudinal analysis of a large public hospital primary care office-based buprenorphine patient registry confirmed that unobserved buprenorphine induction appears a safe, acceptable, feasible, and logistically simple approach to buprenorphine induction. This is in contrast to current US treatment guidelines and buprenorphine product labeling, all of which specify observed induction. Overall, a low threshold office-based treatment paradigm relying on weekly or less frequent follow-up and not requiring additional psychosocial counseling was compatible with robust long-term treatment retention among a primarily heroin dependent, underserved patient population. Continued efforts to expand access to low-barrier buprenorphine maintenance are warranted. Future updates to US treatment guidelines should consider unobserved buprenorphine induction as an accepted standard of care.
